# Hematological Parameters to Predict the Severity of Hyperemesis Gravidarum and Ketonuria

**DOI:** 10.1055/s-0042-1743101

**Published:** 2022-04-11

**Authors:** Mehmet Musa Aslan, Mustafa Taner Yeler, İsmail Bıyık, Hilal Uslu Yuvacı, Arif Serhan Cevrioğlu, Selcuk Özden

**Affiliations:** 1 Department of Obstetrics and Gynecology, Sakarya Training and Research Hospital, School of Medicine, Sakarya University, Sakarya, Turkey; 2 Department of Biochemistry, Muş State Hospital, Muş, Turkey; 3 Department of Obstetrics and Gynecology, Kütahya Health Sciences University, Kütahya, Turkey; 4 Sakarya University, School of Medicine, Sakarya Training and Research Hospital, Department of Obstetrics and Gynecology, Sakarya, Turkey

**Keywords:** hyperemesis gravidarum, ketonuria, hematological parameters, hiperêmese gravídica, cetonúria, parâmetros hematológicos

## Abstract

**Objective** Hyperemesis gravidarum (HG) is a pregnancy complication that can progress with persistent nausea and vomiting. The aim of the present study is to evaluate the relationship between hematological parameters and HG.

**Method** A total of 532 pregnant women with HG who were admitted to the Department of Obstetrics and Gynecology between March 2019 and February 2021, and 534 healthy pregnant women with characteristics similar to those of the case group were included in the study. The hematological parameters of both groups were compared. In addition, the hematological parametersof patients with HG according to the severity of ketonuria were compared.

**Results** The mean age of the HG group ( *n* = 532) was 26.3 ±  4.1 years, and that of the control group ( *n* = 534) was 25.9 ±  4.8 years. Among patients with HG, 46% ( *n* = 249) had ketone (+), 33% ( *n* = 174), ketone (++), and 21% ( *n* = 109), ketone (++ + ). The neutrophil-to-lymphocyte ratio (NLR) and platelet-to-lymphocyte ratio (PLR) were higher in the HG group than in the control group: 3.8 (2.8–5.8)/3.2 (2.6–4.0); *p* < 0.001; and 135.2 ±  30.4/108.9 ±  62.2; *p* < 0.001 respectively. The neutrophil count, NLR, and PLR were higher in the group with ketone (++ + ) than in the groups with ketone (+) or ketone (++): 7.6 ±  1.9/5.5 ±  2.4; *p* < 0.001; 3.8(2.8–4.6)/2.9(2.3–3.6); *p* < 0.001; and 149.9 ±  48.0/135.9 ±  65.7; *p* < 0.001 respectively. The mean corpuscular hemoglobin (MCH) level, the NLR, and the PLR were identified as independent predictors of the presence of HG and the level of ketone positivity in HG patients.

**Conclusion** The NLR and PLR were high in patients with HG, suggesting the its inflammatory activity. They may be important markers associated with the presence and severity of HG.

## Resumo

**Objetivo** A hiperêmese gravídica (HG) é uma complicação da gravidez que pode evoluir com náuseas e vômitos persistentes. O objetivo deste estudo é avaliar a relação entre os parâmetros hematológicos e a HG.

**Método** Foram incluídas neste estudo 532 gestantes com HG internadas no Departamento de Obstetrícia e Ginecologia entre março de 2019 e fevereiro de 2021, e 534 gestantes saudáveis com características semelhantes às do grupo de caso. Os parâmetros hematológicos foram comparados entre gestantes com e sem HG. Além disso, os parâmetros hematológicos foram comparados entre as pacientes com HG de acordo com a gravidade da cetonúria.

**Resultados** A média de idade do grupo GH ( *n* = 532) foi de 26,3 ±  4,1 anos, e a do grupo de controle ( *n* = 534) foi de 25,9 ±  4,8 anos. Entre as pacientes com HG, 46% ( *n* = 249) tinham cetona(+), 33% ( *n* = 174), cetona(++), e 21% ( *n* = 109), cetona(+ + +). A razão de neutrófilos para linfócitos (RNL) e a razão de plaquetas para linfócitos (RPL) foram maiores no grupo HG do que no grupo de controle: 3,8 (2,8–5,8)/3,2 (2,6–4,0); *p* < 0,001; e 135,2 ±  30,4/108,9 ±  62,2; *p* < 0,001, respectivamente). A contagem de neutrófilos, a RNL e a RPL foram maiores no grupo com cetona(++ + ) do que nos grupos com cetona(+) e cetona(++): 7,6 ±  1,9/5,5 ±  2,4; *p* < 0,001; 3,8 (2,8–4,6)/2,9 (2,3–3,6); *p* < 0,001; e 149,9 ±  48,0/135,9 ±  65,7; *p* < 0,001, respectivamente. O nível médio de hemoglobina corpuscular (MHC), a RNL e a RPL foram identificados como preditores independentes da presença de HG e do nível de positividade de cetona em pacientes com HG.

**Conclusão** A RNL e RPL estavam elevadas em pacientes com HG, o que sugere a sua atividade inflamatória. Elas podem ser marcadores importantes associados à presença e à gravidade da HG.

## Introduction

Nausea and/or vomiting occur in ∼ 50% to 80% of pregnant women in the first trimester, and have various negative effects. 1 Hyperemesis gravidarum (HG) is the medical term for severe nausea and vomiting during pregnancy. It may progress with excessive nausea, vomiting, dehydration, ketosis, electrolyte and acid-base imbalance, and sometimes hepatic and renal failure, leading to weight loss (> 5% of body weight). 2 It is is a serious complication of pregnancy, with a frequency of 0.3% to 3%. 3 It typically starts at 4 and 8 weeks of gestation and lasts until weeks 14 to 16. 1 4

Although the underlying cause are not precisely known, it is thought that factors such as high serum levels of steroid hormones, high serum concentrations of human chorionic gonadotropin (hCG), allergens, genetic predisposition, metabolic disorders, hepatic dysfunction, gastrointestinal dysfunction, and neurotic and psychosomatic disorders contribute to the etiology. 3 5 In addition, studies 6 7 have shown that *Helicobacter pylori* could increase the risk of developing HG. In particular, the cytotoxin-associated gene A (CagA) toxin is an important *H. pylori* virulence factor associated with a greater inflammatory response. 8 The role of inflammation in the pathogenesis of HG cannot be adequately explained with current data. Proinflammatory cytokines and inflammatory markers such as interleukin-6 (IL-6) and tumor necrosis factor alpha (TNF-α) have been found to be elevated in HG patients. 9 As metabolic disorders may cause HG, abnormalities in hematological and biochemical parameters may be associated with nausea and vomiting during pregnancy.

In recent studies, 27 28 various hematological parameters have been used to evaluate the inflammatory status of different diseases. The platelet-to-lymphocyte ratio (PLR) and the neutrophil-to-lymphocyte ratio (NLR) are valuable markers that can be obtained from the complete blood count (CBC) at a low cost, with ease and efficiency. Hematological parameters such as the NLR and PLR have been shown to reflect the inflammatory burden and disease activity in several diseases, including ulcerative colitis, spontaneous bacterial peritonitis, malignancies, and cardiovascular diseases. 10 11 12 Hyperemesis gravidarum is a disease that requires hospitalization and affects the psychological and physical health of patients, 13 Its diagnosis is still primarily clinical, and any marker that can be used to predict disease severity may be important. It remains unclear whether hematological parameters are independent markers of the presence and severity of HG.

In the present study, we aimed to investigate the diagnostic value of hematological parameters such as the NLR and PLR in HG patients and their relationship with disease severity.

## Methods

The present is a single-center retrospective study in which the data of 532 pregnant women with HG admitted to our Gynecology and Obstetrics Clinic between March 2019 and February 2021 were analyzed. A total of 534 pregnant women who had no complaints and were age-matched were included as the control group. Both the case and control groups consisted of pregnant women between the ages of 18 and 35 years who were between the 6th and 13th weeks of pregnancy, with positive fetal heartbeat, and gravida 1. All abdominal ultrasonography findings of the study sample were normal. Pregnant women with persistent vomiting with more than 4 episodes a day, ketone positivity in the urine, and 5% weight loss since the beginning of pregnancy were diagnosed as having HG. Patients with any other metabolic or infectious diseases causing nausea, multiple pregnancies, trophoblastic diseases, history of any systematic disease (such as diabetes mellitus, hypertension, and thyroid diseases), psychiatric disorders, any inflammatory disease, use of antiemetics, smoking habits, or alcohol consumption were excluded from the study. All data were retrieved from an electronic medical system, using a specific diagnostic code for HG of the International Classification of Diseases (ICD). The age, height, and weight of the patients were recorded. The body mass index (BMI) was calculated by dividing the weight in kilograms by the square of the height in meters. The study was approved by the Sakarya University Ethics Committee (under number 329), and was conducted in accordance with the guidelines of the Helsinki Declaration.

All blood samples were collected by drawing 5 mL of blood from the antecubital vein without the use of anticoagulants on the day of admission. The CBC values were recorded for each patient. All CBC analyses were performed in our hospital's hematology laboratory using the same Beckman Coulter Gen-S automated analyzer (Brea, CA, United States) for all samples. All CBC parameters, including basophil, eosinophil, hematocrit, hemoglobin (Hb), lymphocyte, mean corpuscular hemoglobin (MCH), mean corpuscular hemoglobin concentration (MCHC), mean cell volume (MCV), monocyte, mean platelet volume (MPV), neutrophil, plateletcrit (PCT), platelet distribution width (PDW), platelet (PLT), red blood cell (RBC), red cell distribution width (RDW), and white blood cell (WBC) values were obtained from the medical records. The NLR was calculated from the differential count by dividing the absolute neutrophil count by the absolute lymphocyte count, and the PLR was calculated by dividing the platelet count by the number of lymphocytes. Ketone levels were analyzed in spot urine samples and classified as (+), (++), or (++ + ). The CBC parameters were compared between both groups. Then, the HG group was separated according to their ketone positivity, and their CBC parameters were compared.

All statistical tests were performed using the Statistical Package for the Social Sciences (IBM SPSS Statistics for Windows, IBM Corp., Armonk, NY, United States). The Kolmogorov-Smirnov test was used to analyze the normality of the data. The continuous data were expressed as means ± standard deviations (SDs), and the categorical data, as percentages. The Chi-squared test was used to assess the differences regarding the categorical variables between groups. The Student *t* -test or the Mann-Whitney U test was used to compare unpaired samples, as needed. The relationship among parameters was assessed by the Pearson or Spearman correlation analysis, according to the normality of the data. Univariate and multivariate logistic regression analyses were used to identify independent variables for HG and ketone severity. The independent variables in the univariate analysis were age, Hb level, RDW, MCH level, MPV, WBC level, neutrophil level, lymphocyte level, NLR, and PLR. After the univariate analysis, significant variables were selected for the multivariate logistic regression analysis using the stepwise method. The results of the univariate and multivariate regression analyses are presented as odds ratios (ORs) with 95% confidence intervals (95%CIs). All independent variables in the logistic regression were tested for multicollinearity. If the variance inflation factor (VIF) exceeded 3.0, the variable was considered collinear. Statistical significance was set at two-sided *p* < 0.05.

## Results

**Fig. 1:**
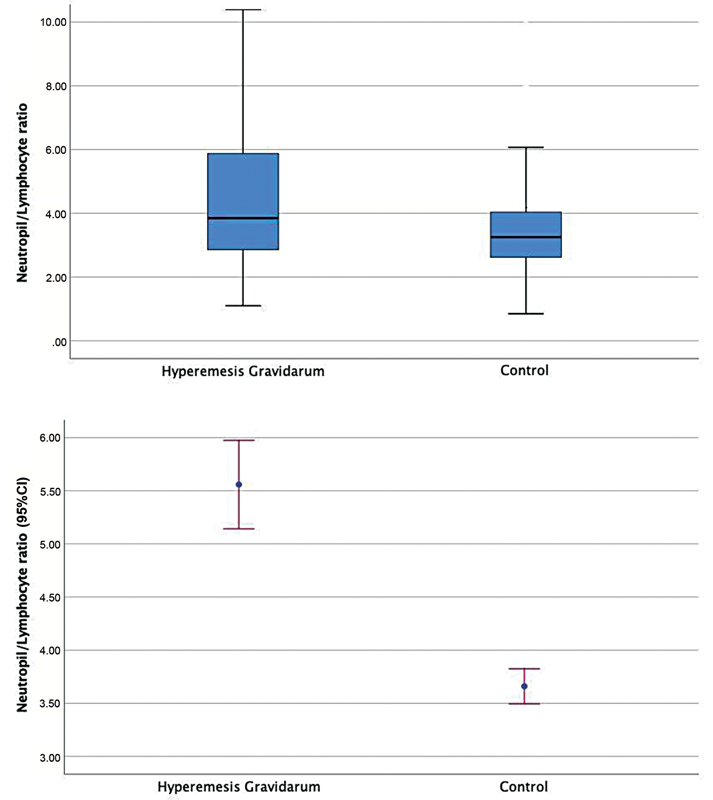
Comparison of the neutrophil-to-lymphocyte ratio (NLR) of patients with hyperemesis gravidarum and the control group.

Table 1 shows the clinical and demographic characteristics of the 532 patients with HG and the 534 age-matched controls in the study. There was no statistically significant difference between the groups in terms of age and BMI. The CBC parameters of the groups were compared, and RBC, hematocrit, Hb, and PLT levels were found to be significantly higher in the HG group than in the control group (4.5 ±  0.5/4.3 ±  0.4; *p* < 0.001; 38.6 ± 4.2/36.0 ±  3.8; *p* < 0.001; 12.9 ±  1.5/12.1 ±  1.4; *p* < 0.001; and 284.2 ±  63.7/207.3 ±  56.4; *p* = 0.033 respectively). Likewise, the MCH, MCV, and MPV values were significantly higher in the HG group than in the control group (28.6 ±  2.6/27.9 ±  2.7; *p* < 0.001; 85.5 ±  5.9/83.0 ±  6.2; *p* < 0.001; and 8.3 ±  1.5/8.0 ±  1.5; *p* = 0.005, respectively). The MCHC was significantly lower in the HG group than in the control group (33.4 ±  1.6/33.6 ±  1.3; *p* = 0.039). There was no difference in the PCT level between the groups. The PDW was significantly higher in the HG group than in the control group: 20.1 ±  1.2 and 19.5 ±  1.4; *p* < 0.001 respectively. The WBC and neutrophil levels were significantly higher in the HG group than in the control group: 12.3 ±  4.2/11.4 ±  4.2; *p* = 0.001; and 9.6 ±  4.5/6.6 ±  2.3; *p* < 0.001 respectively). There was no difference between the groups in terms of basophil and eosinophil levels. The lymphocyte level was higher in the HG group than in the control group: 2.1 ±  0.7 and 1.9 ±  0.6; *p* < 0.001 respectively). The NLR and PLR were also higher in the HG group than in the control group: 3.8(2.8–5.8)/3.2(2.6–4.0); *p* < 0.001; and 135.2 ±  30.4/108.9 ±  62.2; *p* < 0.001 respectively) ( Fig. 1 ). The HG patients were categorized according to their ketone levels; there were 249 (46%) patients with ketone (+), 174 (33%) with ketone (++) was, and 109 (21%) with ketone (++ + ). Patients with ketone (++ + ) were compared with patients with ketone (+) and ketone (++); the neutrophil count, NLR, and PLR were found higher in the ketone (+++) group than ketone(+) and ketone(++); groups (3.8(2.8–4.6) vs 2.9 (2.3–3.6), *p* <0.001 for NLR; and 149.9 ± 48.0 vs 135.9 ± 65.7, *p* <0.001 for PLR rescpectively) ( Table 2 ).

**Table 1:** Demographic and clinical data of the study sample

Clinical characteristics	Control group ( *n* = 534)	HG group ( *n* = 532)	*p* -value
Age (years)	25.9 ± 4.8	26.3 ± 4.1	0.212
BMI (kg/m ^2^ )	24.79 ± 3.17	23.12 ± 3.48	0.488
Ketone positivity	−	1.7 ± 0.7	< 0.001
RBC	4.3 ± 0.4	4.5 ± 0.5	< 0.001
Hb (g/dL)	12.1 ± 1.4	12.9 ± 1.5	< 0.001
Hematocrit (%)	36.0 ± 3.8	38.6 ± 4.2	< 0.001
RDW (%)	13.2 ± 1.9	13.1 ± 2.0	0.213
MCH (pg)	27.9 ± 2.7	28.6 ± 2.6	< 0.001
MCHC (g/dL)	33.6 ± 1.3	33.4 ± 1.6	0.039
MCV (fL)	83.0 ± 6.2	85.5 ± 5.9	< 0.001
MPV (fL)	8.0 ± 1.5	8.3 ± 1.5	0.005
PLT (/mm ^3^ ×10 ^3^ )	207.3 ± 56.4	284.2 ± 63.7	0.033
PCT (%)	0.1 ± 0.04	0.1 ± 0.05	0.714
PDW (%)	19.5 ± 1.4	20.1 ± 1.2	< 0.001
WBC (/mm ^3^ ×10 ^3^ )	11.4 ± 4.2	12.3 ± 4.2	0.001
Neutrophil (/mm ^3^ ×10 ^3^ )	6.6 ± 2.3	9.6 ± 4.5	< 0.001
Basophil (/mm ^3^ ×10 ^3^ )	0.1(0.0–0.1)	0.1(0.0–0.1)	0.489
Eosinophil (/mm ^3^ ×10 ^3^ )	0.1(0.0–0.1)	0.1(0.0–0.1)	0.646
Lymphocyte (/mm ^3^ ×10 ^3^ )	1.9 ± 0.6	2.1 ± 0.7	< 0.001
Monocyte (/mm ^3^ ×10 ^3^ )	0.6 ± 0.2	0.5 ± 0.2	< 0.001
Neutrophil-to-lymphocyte ratio	3.2(2.6–4.0)	3.8(2.8–5.8)	< 0.001
Platelet-to-lymphocyte ratio	108.9 ± 62.2	135.2 ± 30.4	< 0.001
Ketone positivity, n(%)			
0	534(100)	0(0)	
+	0(0)	249(46)	
+ +	0(0)	174(33)	
+ ++	0(0)	109(21)	

Abbreviations: BMI, Body mass index; Hb, hemoglobin; HG, hyperemesis gravidarum; MCH, mean corpuscular hemoglobin; MCHC, mean corpuscular hemoglobin concentration; MCV, mean cell volume; MPV, mean platelet volume; PCT, plateletcrit; PDW, platelet distribution width; PLT, platelet; RBC, red blood cell; RDW, red cell distribution width; WBC, white blood cell.

Note: Values expressed as means ± standard deviations, or as medians(ranges), unless otherwise specified.

**Table 2:** Comparison of patients with hypermesis gravidarum according to ketone positivity

Clinical characteristics	Ketone +/+ + ( *n* = 423)	Ketone +++ ( *n* = 109)	*p* -value
Age (years)	26.1 ± 4.0	26.9 ± 4.3	0.111
RBC	4.5 ± 0.4	4.4 ± 0.6	0.339
Hb (g/dL)	13.0 ± 1.3	12.5 ± 1.8	0.014
Hematocrit (%)	38.7 ± 3.9	37.9 ± 5.1	0.106
RDW (%)	13.1 ± 2.0	12.9 ± 1.8	0.228
MCH (pg)	28.8 ± 2.5	28.0 ± 2.8	0.004
MCHC (g/dL)	33.5 ± 1.6	33.0 ± 1.7	0.003
MCV (fL)	85.7 ± 5.8	84.7 ± 6.3	0.116
MPV (fL)	8.3 ± 1.5	8.4 ± 1.5	0.699
PLT (/mm ^3^ ×10 ^3^ )	270.0 ± 57.2	298.5 ± 53.3	0.404
PCT (%)	0.1 ± 0.05	0.1 ± 0.04	0.637
PDW (%)	19.4 ± 1.3	19.7 ± 1.8	0.153
WBC (/mm ^3^ ×10 ^3^ )	11.4 ± 4.2	11.7 ± 3.9	0.499
Neutrophil (/mm ^3^ ×10 ^3^ )	5.5 ± 2.4	7.6 ± 1.9	< 0.001
Basophil (/mm ^3^ ×10 ^3^ )	0.1(0.0–0.1)	0.0(0.0–0.1)	0.127
Eosinophil (/mm ^3^ ×10 ^3^ )	0.1(0.0–0.1)	0.1(0.0–0.1)	0.609
Lymphocyte (/mm ^3^ ×10 ^3^ )	1.9 ± 0.6	2.0 ± 0.5	0.062
Monocyte (/mm ^3^ ×10 ^3^ )	0.5 ± 0.2	0.5 ± 0.2	0.774
Neutrophil-to-lymphocyte ratio	2.9(2.3–3.6)	3.8(2.8–4.6)	< 0.001
Platelet-to-lymphocyte ratio	135.9 ± 65.7	149.9 ± 48.0	< 0.001

Abbreviations: Hb, hemoglobin; MCH, mean corpuscular hemoglobin; MCHC, mean corpuscular hemoglobin concentration; MCV, mean cell volume; MPV, mean platelet volume; PCT, plateletcrit; PDW, platelet distribution width; PLT, platelet; RBC, red blood cell; RDW, red cell distribution width; WBC, white blood cell.

Note: Values expressed as means ± standard deviations, or as medians(ranges).

Parameters affecting the presence of HG were identified by univariate and multivariate analyses with logistic regression. The age, Hb, RDW, MCH, MPV, WBC, neutrophil, lymphocyte, NLR, and PLR were first evaluated in the univariate analysis. The Hb, MCH, MPV, WBC, neutrophil, lymphocyte, NLR, and PLR were statistically significant in univariate analysis, and they were reevaluated in the multivariate analysis. The Hb, MCH, MPV, WBC, NLR, and PLR were found to be significant independent predictors of the presence of HG (Hb – OR: 1.409; *p* < 0.001; MCH – OR: 0.785; *p* < 0.001; MPV – OR: 1.161; *p* < 0.001; WBC – OR: 0.952; *p* = 0.002; NLR – OR: 0.817; *p* < 0.001; and PLR – OR: 1.291. *p* < 0.001). However, parameters that were thought not to affect the development of HG or did not reach statistical significance between the groups were not included in the regression model ( Table 3 ).

**Table 3:** Univariate and multivariate logistic regression analyses of the risk factors associated with the presence of hyperemesis gravidarum

Variable	Univariate			Multivariate		
	OR	95%CI	*p* -value	OR	95%CI	*p* -value
Age	1.011	0.986–1.037	0.392			
Hemoglobin	1.440	1.321–1.570	< 0.001	1.409	1.280–1.552	< 0.001
RDW	0.973	0.917–1.033	0.365			
MCH	1.098	1.051–1.147	< 0.001	0.785	0.701–0.881	< 0.001
MPV	1.068	1.046–1.090	< 0.001	1.161	1.104–1.221	< 0.001
WBC	0.953	0.927–0.979	0.001	0.952	0.923–0.982	0.002
Neutrophil	0.952	0.939–0.965	< 0.001	0.991	0.971–1.011	0.370
Lymphocyte	1.063	1.046–1.081	< 0.001	0.995	0.964–1.026	0.733
NLR	0.819	0.777–0.862	< 0.001	0.817	0.772–0.864	< 0.001
PLR	1.081	1.003–1.165	0.042	1.291	1.173–1.420	< 0.001

Abbreviations: 95%CI, 95% confidence interval; MCH, mean corpuscular hemoglobin; MPV, mean platelet volume; NLR, neutrophil-to-lymphocyte ratio; OR, odds ratio; PLR, platelet-to-lymphocyte ratio; RDW, red cell distribution width; WBC, white blood cell.

Parameters affecting the level of ketone positivity were identified through univariate and multivariate analyses with logistic regression. The MCH, NLR, and PLR were found to be significant independent predictors of the level of ketone positivity in HG patients (MCH – OR: 0.889; *p* = 0.003; NLR – OR: 1.111; *p* = 0.033; PLR – OR: 0.995; *p* = 0.035) ( Table 4 ).

**Table 4:** Univariate and Multivariate logistic regression analyses of the risk factors associated according to ketone positivity levels

Variable	Univariate			Multivariate		
	OR	95%CI	*p* -value	OR	95%CI	*p* -value
Age	1.044	0.992–1.099	0.096	1.042	0.989–1.099	0.124
Hemoglobin	0.821	0.718–0.938	0.004	0.905	0.772–1.059	0.213
RDW	0.934	0.836–1.044	0.228			
MCH	0.898	0.833–0.968	0.005	0.889	0.824–0.960	0.003
MCV	0.973	0.940–1.007	0.117			
MPV	1.026	0.900–1.171	0.699			
WBC	1.017	0.968–1.068	0.498			
Neutrophil	0.992	0.968–1.017	0.522			
Lymphocyte	0.990	0.962–1.020	0.511			
NLR	1.100	0.998–1.211	0.054	1.111	1.007–1.226	0.033
PLR	0.996	0.992–1.000	0.074	0.995	0.991–0.999	0.035

Abbreviations: 95%CI, 95% confidence interval; MCH, mean corpuscular hemoglobin; MCV, mean cell volume; MPV, mean platelet volume; NLR, neutrophil-to-lymphocyte ratio; OR, odds ratio; PLR, platelet-to-lymphocyte ratio; RDW, red cell distribution width; WBC, white blood cell.

The Pearson or Spearman correlation analysis was used to evaluate the relationship between ketone positivity and CBC parameters. A statistically significant positive correlation was found between the level of ketone positivity and Hb, neutrophil, NLR, and PLR (r = 0.290; *p* < 0.001; r = 0.276; *p* < 0.001; r = 0.664; *p* < 0.001; and r = 0.590; *p* < 0.00 respectively) ( Table 5 ).

**Table 5:** Pearson/Spearman correlation coefficients regarding complete blood count parameters and ketone positivity levels in all patients

	Pearson/Spearman	Hb	MCV	Neutrophil	PDW	WBC	NLR	PLR
**Ketone**	***r***	0.250	0.163	0.276	-0.171	-0.102	0.664	0.590
	***p***	< 0.001	< 0.001	< 0.001	< 0.001	< 0.001	< 0.001	<0.001

Abbreviations: Hb, hemoglobin; MCV, mean cell volume; NLR, neutrophil-to-lymphocyte ratio; PDW, platelet distribution width; PLR, platelet-to-lymphocyte ratio; WBC, white blood cell.

## Discussion

In the present study, we aimed to investigate the diagnostic value of hematological parameters in HG patients and their relationship with disease severity. Our main findings were as follows: 1) the NLR and PLR were higher in the HG group than in the control group; 2) the NLR and PLR were higher in HG patients with ketone (++ + ), indicating greater disease severity compared with that of patients with ketone (+) or ketone (++); 3) the MCH, NLR, and PLR were independent predictors of the presence of HG and of the level of ketone positivity in HG patients; and 4) a statistically significant positive correlation was found between the ketone positivity level and the Hb, neutrophil, NLR, and PLR.

Severe vomiting and nausea are characteristic in HG, leading to malnutrition, electrolyte imbalance, and disruption in biochemical parameters, and generally requiring hospitalization. 14 15 Although the etiology of HG is not fully known, psychological factors, hormonal changes, abnormal gastrointestinal motility, *H. pylori* , vitamin/mineral deficiencies, changes in the autonomic nervous system, changes in the lipid profile, genetic factors, and immunological regulation disorders have been implicated in HG. 3 15 16 However, the net effect of any factor could not be determined. Nevertheless, active or chronic *H. pylori* infection is observed more frequently in HG patients compared with asymptomatic pregnant women, and certain parameters such as inflammation-related CRP and IL-6 are higher in HG patients, suggesting that inflammation may play a role in the pathogenesis of this disease. 6 9

In addition to the classic markers of inflammation, the NLR and PLR have been investigated 10 11 12 17 18 as inflammatory markers in recent years. The basis of these studies is the physiological response of leukocytes to stimuli, the increase in the number of neutrophils, and the decrease in the lymphocyte count accompanying neutrophilia. 17 The NLR has been shown to play a prognostic role in various disease groups, such as infectious diseases, metabolic syndrome, chronic obstructive pulmonary disease, end-stage renal disease, subdural hemorrhage, Behcet disease, malignancy, and diseases of the cardiovascular system. 10 11 12 The PLR has been reported to be an independent predictor of reduced survival, with a negative prognostic value in gynecological and hepatobiliary system malignancies. 18

Various studies have been conducted to investigate the diagnostic importance of hematological system markers in HG patients. In a prospective study by Kurt et al., 19 the NLR and high-sensitivity CRP level were found to be significantly higher in the HG group. In a retrospective study by Tayfur et al. 20 including 433 pregnant women, the authors found that inflammatory markers such as the NLR, PLR, and PCT level were significantly higher in HG patients. In a prospective study by Beyazit et al. 21 with 112 patients, the PLR and NLR were found to be higher in patients with HG, and the NLR was found to be correlated with the CRP level. 21 In a prospective study including 355 pregnant women, the WBC, NLR, PLR, and RDW were found to be higher in the HG group, and even in the late second trimester of pregnancy, they remained high in patients with HG. 22 A study by Çintesun et al. 23 demonstrated that the NLR and PLR were effective markers in HG. In agreement with the literature, the NLR and PLR were high in patients with HG in the present study. In addition, we found that the NLR and PLR were independent predictors of the presence and severity of HG. In comparison with other studies, the present involved a larger sample.

In patients with HG, increased hemoconcentration can be expected due to vomiting. However, Sari et al. 24 found that the Hb and hematocrit levels did not change in HG patients. In the present study, the Hb and hematocrit levels were high in patients with HG. In addition, we identified the level of Hb as an independent predictor of the presence of HG. The lymphocyte count tends to be higher in pregnant women with HG. 9 However, some studies 20 24 have reported no changes in the lymphocyte count in HG patients. In the present study, the lymphocyte count was higher in the HG group.

The PDW, PCT level, and MPV reflect changes in the PLT volume, and are thought to indicate PLT activation. 25 Beyazit et al. 21 found that the PDW and MPV did not differ significantly between HG patients and controls. 21 Tayfur et al. 20 found that the PCT level was significantly higher in HG patients than in healthy pregnant controls. In the present study, although there was no difference in the PCT level, the PDW and MPV were high in HG patients. In addition, the MPV was identified as an independent marker in the diagnosis of the disease.

Ketonuria is a parameter used in the diagnosis of severe HG. Hypokalemia, hypochloremic metabolic alkalosis, and ketosis due to low calorie intake can occur in pregnant women. In a study 26 comparing patients with and without HG, higher ketonuria was observed in the HG group, and prolonged hospital stay was associated with higher ketonuria. In a study 23 investigating the relationship between ketonuria and hematological parameters, no marker other than the RDW was correlated with the degree of ketonuria.

In the present study, the patients were grouped according to their ketone positivity level, and hematological parameters were compared. We found that, as the severity of HG increased, the neutrophil count, NLR, and PLR were significantly higher in patients with ketone (++ + ) than in patients with ketone (++) or ketone (+) in the urine. In contrast to other studies, we showed that the MCH level, NLR, and PLR were independent predictors of the presence and severity of HG. In addition, there was a positive correlation between the ketone positivity level and the Hb, neutrophil, NLR, and PLR parameters.

The present study has several limitations. First, it was a single-center and retrospective study. Second, the Pregnancy Unique-Quantification of Emesis (PUQE) scoring system and the Rhodes index, which enable a more objective evaluation of HG, were not calculated. Third, other inflammatory markers such as the CRP, sedimentation, and IL-6 were not included in the analysis, as they were not measured in all patients.

## Conclusion

In conclusion, the results of the present study have demonstrated the association of hematological inflammatory parameters with HG, which may be used to determine disease severity. The NLR and PLR were high in patients with HG, which indicated the inflammatory nature of pregnancy nausea and vomiting. The present study has shown that the NLR and PLR may be used as markers of the inflammatory burden in HG patients. The addition of hematological markers, especially the NLR and PLR, to scoring systems could enable a more objective evaluation of the disease. Further studies on the relationship between HG and inflammation with larger prospective samples are needed.
